# Incidents Connected with the Trial of Professor Webster

**Published:** 1894-01

**Authors:** Lester Noble

**Affiliations:** Springfield, Mass.


### INCIDENTS CONNECTED WITH THE TRIAL OF PROFESSOR WEBSTER.[1]

BY DR. LESTER NOBLE, SPRINGFIELD, MASS.

[On November 23 1849, Professor John W. Webster, of the Harvard Medical School, murdered Dr. George Parkman, a retired physician of Boston. The trial of Professor Webster in March, 1850, was at that time the most noted murder trial ever held in Massachusetts. The following brief account was given at the Union Meeting of the Connecticut Valley and Connecticut State Dental Societies, held in Hartford, May 16, 17 and 18, 1893, by Dr. Lester Noble, of Springfield, who with Dr. Keep was a witness for the prosecution.]

At the time the murder was committed I was a student in the Baltimore College of Dental Surgery, and was summoned as a witness for the prosecution. The crime was committed on Friday in the Medical College, Boston, after the students had all left. On the following Friday, Professor Webster was arrested. In the mean time he endeavored to dispose of the body; he cut it up, burned the head and large portions of the body in the laboratory furnace, hiding other parts in various places. The destruction was so effectual that the most important evidence for conviction was the identification of the artificial teeth. The question had been raised whether Dr. Keep and myself could surely identify these teeth, after they had been subjected to such a heat, as those which we had made for Dr. Parkman, and which had been worn by him. This evidence was of vital importance, for if we could not prove that the jaw and teeth were Dr. Parkman's the prosecution *could not prove his death.*

---

[1] Read before the Connecticut State Dental Association, Hartford, May 16, 17, 18, 1893.

Further, if they failed to prove the death of Dr. Parkman it was impossible to demonstrate that Dr. Webster or any one else had killed him, hence the importance of Dr. Keep's and my testimony in the case.

At this time there was a supply of manufactured teeth on the market, yet they were all single teeth, and it was considered altogether more desirable for a dentist to manufacture the artificial teeth used in his practice if he were competent to do so.

There were found among the charred human remains in the furnace an upper and a lower set of *block* teeth; the gold plates were melted to nuggets, the lower teeth somewhat injured, but the full upper set in three blocks was perfect, except being slightly bent. I will now state Dr. Keep's mode of manufacturing artificial teeth, to show how it was possible to identify them. After the plates had been fitted, and a set of teeth carved out of wax, the exact size and style required, moulds were made. Everything in plaster connected with the case was carefully kept and labelled in a box by itself, and this numbered and entered in the catalogue.

When the teeth were moulded they were baked slightly, just enough to give them strength, and yet be easily cut with a little twist drill held by the thumb and finger. We made four or six holes in a front block of six teeth and three or four in a side block of four teeth, and they must be absolutely parallel in both directions. The enamel was then placed on, and the gum color, inside as well as outside. This was a specialty of Dr. Keep's. The teeth were again baked until properly vitrified. This accomplished, a wood cylinder was fitted to each hole, and the gold wire to be used for pins, soldered on the gold plate, which fitted accurately this cylinder. With moderate pressure, the central block of six teeth would go to its exact position on the plate; the side blocks were put in place in the same manner. It may appear a difficult task to place these pins on the plate with such exactness that the block would go down without binding, but it could always be accomplished. When the set was wet or even moistened the teeth were perfectly tight; after a thorough drying they could at any time be removed.

Dr. Keep made a set of teeth for Dr. Parkman soon after I entered his office as a student. This was about a year before the advent of central air-chambers, and each plate was held in place by spiral springs attached to the artificial gum above the bicuspids, by a hole drilled through the block.

With Dr. Parkman's teeth I had more than a first acquaintance. He was a very nervous man, and occasionally would take out his plates and put them in his coat-tail pocket, and soon he would forget them, and when he sat down the teeth would suffer more or less, necessitating repairs. I must have repaired those plates at least half a dozen times. The lower jaw had on the left side, the cuspid, the lateral incisor, and the root of the first bicuspid; on the right side, the cuspid and the roots of the first and second bicuspids. The gold plate went over these roots on both the right and left side and around the three teeth which were entire. All the teeth from the cuspid back on the left side were in one block, and those from the first bicuspid on the right side were also in one block. There was also the small block of three incisors. Any dentist will see that it was an extraordinary shaped plate, requiring a very unusual block to fit it. Dr. Parkman insisted on having the plate made over these roots; when Dr. Keep objected Dr. Parkman said, "I pay the bills; I want you to try it, and do the best you can!" The lower teeth were ground upon the inside to make room for his tongue, thus grinding off a portion of the gum color. I was present when Dr. Keep ground them, with an old copper cent for a wheel, and the addition of water and emery. The teeth found in the furnace fitted exactly into our moulds, and they plainly showed the marks of the grinding, and the holes where the spiral springs had been inserted. Further, there was no mistaking the peculiar shade of gum color and of the enamel of the teeth made from Dr. Keep's secret recipes. There was in the Dr. George Parkman box of models, duly marked and dated, a model made from a wax impression of the lower jaw, showing the number and position of the teeth and roots which were there when the teeth were made. The charred and broken lower jaw had them in the same number and same position as the plaster model, made three years before. If there had been found just one tooth or root of a different class from those the model showed Dr. Parkman to have had, it would have been a powerful argument for the defence, but, as it was, the identification was complete. Professor Webster was convicted, and hung in the following August. Before his death he made a confession of the murder.

